# Detection of trachoma using machine learning approaches

**DOI:** 10.1371/journal.pntd.0010943

**Published:** 2022-12-07

**Authors:** Damien Socia, Christopher J. Brady, Sheila K. West, R. Chase Cockrell

**Affiliations:** 1 Division of Surgical Research, Department of Surgery, Larner College of Medicine, University of Vermont, Burlington, Vermont, United States of America; 2 Division of Ophthalmology, Department of Surgery, Larner College of Medicine, University of Vermont, Burlington, Vermont, United States of America; 3 Dana Center for Preventive Ophthalmology, Wilmer Eye Institute, Baltimore, Maryland, United States of America; Yale University School of Medicine, UNITED STATES

## Abstract

**Background:**

Though significant progress in disease elimination has been made over the past decades, trachoma is the leading infectious cause of blindness globally. Further efforts in trachoma elimination are paradoxically being limited by the relative rarity of the disease, which makes clinical training for monitoring surveys difficult. In this work, we evaluate the plausibility of an Artificial Intelligence model to augment or replace human image graders in the evaluation/diagnosis of trachomatous inflammation—follicular (TF).

**Methods:**

We utilized a dataset consisting of 2300 images with a 5% positivity rate for TF. We developed classifiers by implementing two state-of-the-art Convolutional Neural Network architectures, ResNet101 and VGG16, and applying a suite of data augmentation/oversampling techniques to the positive images. We then augmented our data set with additional images from independent research groups and evaluated performance.

**Results:**

Models performed well in minimizing the number of false negatives, given the constraint of the low numbers of images in which TF was present. The best performing models achieved a sensitivity of 95% and positive predictive value of 50–70% while reducing the number images requiring skilled grading by 66–75%. Basic oversampling and data augmentation techniques were most successful at improving model performance, while techniques that are grounded in clinical experience, such as highlighting follicles, were less successful.

**Discussion:**

The developed models perform well and significantly reduce the burden on graders by minimizing the number of false negative identifications. Further improvements in model skill will benefit from data sets with more TF as well as a range in image quality and image capture techniques used. While these models approach/meet the community-accepted standard for skilled field graders (i.e., Cohen’s Kappa >0.7), they are insufficient to be deployed independently/clinically at this time; rather, they can be utilized to significantly reduce the burden on skilled image graders.

## Author summary

Trachoma is an infectious disease, experienced primarily in the developing world, and is a leading cause of global blindness. As recent efforts to address the disease have led to a significant reduction in disease prevalence, it has become difficult to train health workers to detect trachoma, due to its rarity; this if often referred to as the “last mile” problem. To address this issue, we have implemented a convolutional neural network to detect the presence of TF in images of everted eyelids. The trained network has comparable performance to trained, but non-expert, human image graders. Further, we found that misclassified images were typically characterized by poor image quality (e.g., blurry, eyelid not in image, etc.), which could be addressed by a standardization of the image acquisition protocol.

## Introduction

Despite intensive worldwide control efforts, trachoma remains the most important infectious cause of vision loss [1] and one of the overall leading causes of global blindness,[2,3] with nearly 125 million people at risk of vision loss [4]. The World Health Organization (WHO) set a goal for global elimination of trachoma as a public health problem by 2020[5], and while this has contributed to a significant reduction in prevalence of the disease, additional strategies will be necessary for global elimination. This is due to the “last mile” problem [6][7–9], which, in essence, describes situations in which the additional application of conventional resources leads to diminishing returns. In this case, the decreasing prevalence of TF makes it more difficult to both train new field graders to detect the disease, and for existing graders to maintain their skills—without ongoing exposure to the clinical sign of TF, graders’ clinical accuracy atrophies [8–10].

The conventional means of disease control has been aggressive surveillance for clinical signs of active disease (key sign: trachomatous inflammation—follicular, TF) and whole district treatment with azithromycin if the prevalence of TF in 1–9-year-olds is 5% or higher. Crucially, when the disease prevalence approaches 5% or less, clinical accuracy tolerances should theoretically be much tighter than when prevalence is high because the decision about continuing treatment (or restarting programs in the face of disease re-emergence) has such important and expensive ramifications. Travel to endemic areas has been used for training and ongoing standardization of graders, but there are increasingly few places with an adequate prevalence of TF to justify the cost and logistical complexity of travel. With the global COVID-19 pandemic, travel has become even more problematic and has further escalated the need for new solutions.

Ophthalmic photography has been used for standardized clinical diagnosis for research purposes in many conditions including trachoma,[11–13] but the utility of imaging at scale in control programs that operate almost exclusively in rural areas of resource-poor countries remains unknown. Additionally, expert-grading of images in a conventional reading-center model is labor-intensive and expensive, and unlike the upfront investment for extensive training and potential travel to certify a skilled grader, these costs would generally be expected to ongoing, per patient, for the life of the program. While we are not aware of a commercial fee schedule for remote grading of trachoma images, in the United States, grading of eye images has a 2022 Medicare allowable charge between $20–28 per patient [14]. While more cost-effective than conventional in-person examination for some eye conditions in the US, this cost is still likely orders of magnitude higher than could be scalable for trachoma control programs [15–18]. As such, there are two distinct, but inter-related efforts, with the goal of facilitating the identification of districts in which population-based interventions are needed to address endemic trachoma: crowdsourcing [19–21], used successfully in our prior work, and artificial intelligence [22]. Previous work conducted by our team using a smartphone-based image capture system (ICAPS) demonstrated that eyelid photography of sufficient quality for non-expert grading can be acquired in a district-level trachoma survey, transmitted from the field via the internet, and graded remotely in a Virtual Reading Center by both an expert grader or by nonexpert crowdsourcing workers at lower cost per image than clinical grading [6,23].

Other groups [22] have utilized artificial neural networks to grade trachoma images and shown that, though this is a promising scalable technique, it would need further development to be utilized as a clinical tool. In this work, we examine the feasibility of augmenting and/or replacing the expert grader/crowdsourcing workflow with an AI. In this study, we utilize state-of-the-art Convolutional Neural Network (CNN) architectures to evaluate and grade the same set of images that were used in the ICAPS study for comparison. We validate our model against previously published data and demonstrate feasibility, but that further standardization of our image capture workflow is needed to render the Artificial Intelligence (AI) model clinically relevant.

## Methods

### Ethics statement

This work was deemed research that does not involve human subjects by the Institutional Review Board at the University of Vermont, and thus exempt from Institutional Review Board review.

### Dataset

The dataset of 2,614 everted upper eyelid images used for this study was collected during a 2019 district level survey in Chamwino, Tanzania as part of the Image Capture and Processing System (ICAPS) development study [6]. For this study we analyzed the set of 2,299 ICAPS images that received the same TF grade from a single international trachoma expert and a single field grader, randomly divided into a training/validation (n = 44 TF) and test set (n = 12 TF). This photo-field concordant grade was considered the “ground truth” for analysis of AI grading. To validate our work, we considered an additional dataset [22] consisting of 1546 total images with n = 421 TF for training and validation, and n = 106 TF for the test set.

### Convolutional neural network classifier

The Convolutional Neural Network (CNN) [24,25], is a type of neural network that is commonly used in image processing [26,27] and is motivated by the biological architecture of the visual cortex. The neural network architectures used in this work were ResNet101 [28] and VGG16 [29], with the number of output classes for each model reduced to two (positive or negative for TF). Class weighting and batch accumulation [30] were utilized for efficient model training. Class weighting adds a weight to the positive class when calculating the loss, causing incorrect predictions for TF-positive images to have a larger impact on model training than incorrect predictions of negative images. Batch accumulation is a method to increase batch size on memory constrained machines, by accumulating the loss function from several batches prior to backpropagation. We used the binary cross-entropy loss function [31], as this is widely-recognized to lead to the most efficient training and most accurate results.

### Data augmentation

We note that out of the approximately 2000 images in the primary dataset, only ~2.5% of the images were positive for TF. Due to the unbalanced nature of the dataset, we examined a variety of oversample ratios to optimize the performance of the chosen networks. Ultimately, we found that oversample ratios greater than 50% (meaning that additional copies of TF-positive images were inserted into the training set such that it resulted in a 2:1 ratio of TF-negative:TF-positive images) did not increase the performance of the network.

Additionally, we utilized several data augmentation techniques on the oversampled images (allowing the copies to be subtly different than their originals): horizontal flipping [32], rotation[33], perspective shift [34,35], and color jitter [36,37]. The horizontal flipping reflects the image across its central axis (changing an image of a left-eye to an image of the right-eye and vice versa). For rotation, the orientation of the image was adjusted by ±15 degrees, inspired by the potential camera-alignments of the data collectors. Perspective shift projects the two-dimensional image onto a three-dimensional plane, simulating image capture from different angles. Color jitter transforms randomly change the hue, saturation, and brightness of the image. During the training, each image transform was applied to the TF-positive images with a probability of 0.5; this probability was applied individually to each transform, allowing for various combinations of data augmentation techniques to be applied to each image. Data augmentation techniques were only applied to the training set. Subsequent to the data augmentation, color-spaces in all images were normalized to match the color distribution of the training set, and all images were resized such that they had an area of 224 x 224 pixels.

### Follicle enhancement

**Fig 1:**
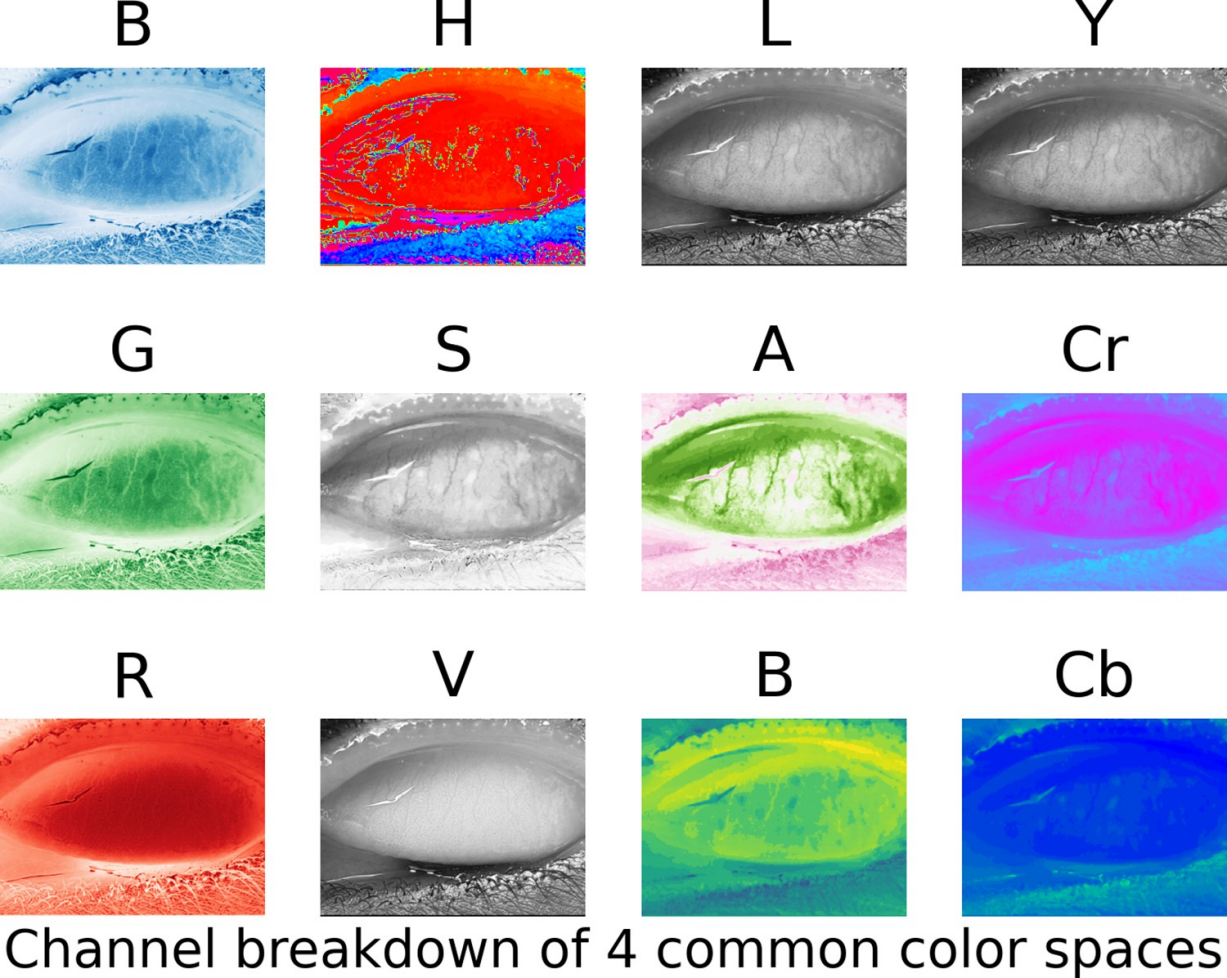
**Color Space Illustration:** Here we present the four color-spaces that were used when developing the initial neural network models.Each color space, represented as column in this image, is defined by a tripled, represented by the rows in this image.

**Fig 2:**
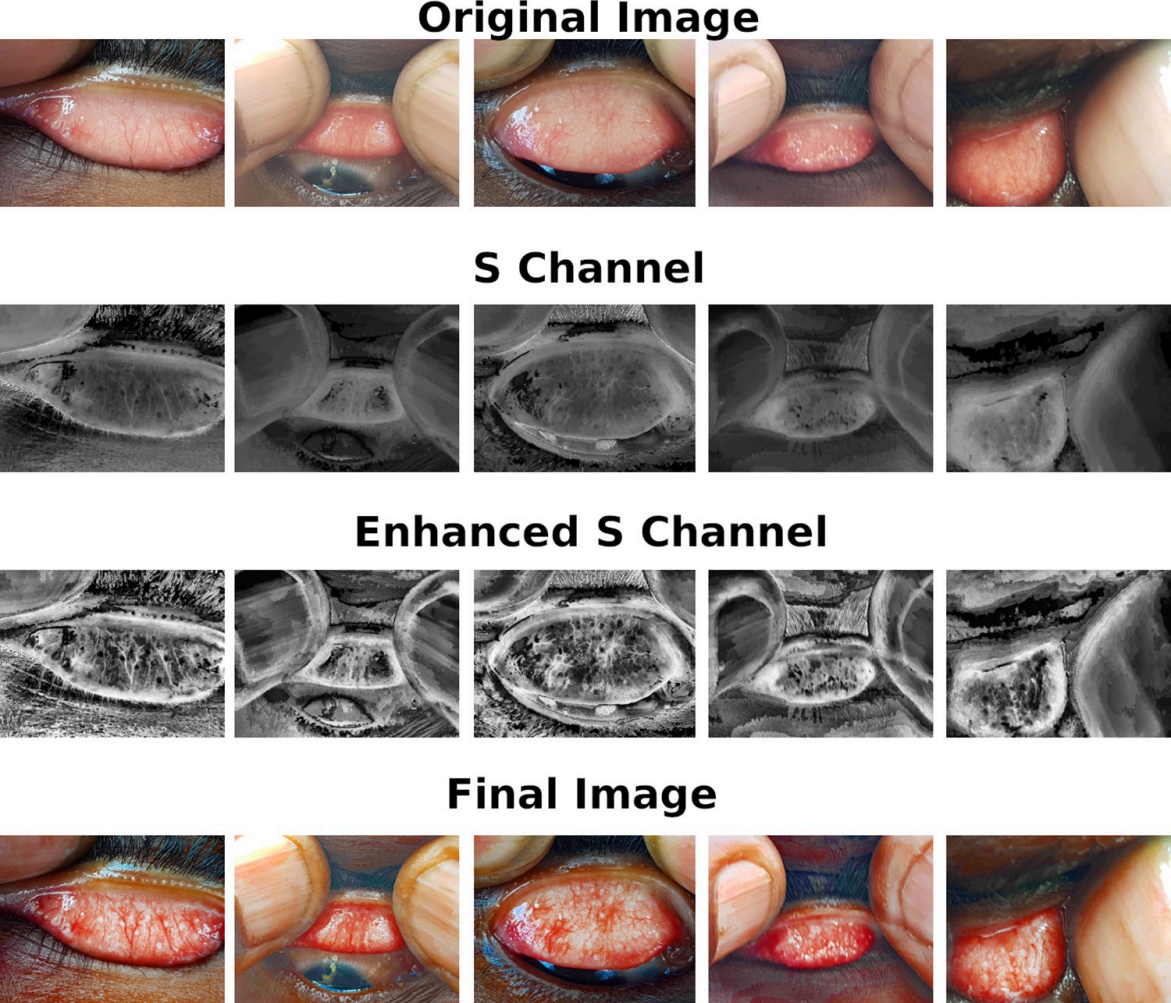
**Follicle Enhancement:** In an attempt to encourage the network to learn the clinical features that identify trachomatous inflammation (i.e., ≥ 5 follicles with ≥ 0.5 mm diameter), we enhanced the contrast of the follicles using the Contrast Limited Adaptive Histogram Equalization algorithm. The results of this on the S channel are shown in the third row and the final image is then shown in the fourth row.

Because the clinical definition of TF is based on the number of follicles ≥ 0.5mm in diameter, a method was devised to improve the contrast between the follicles and the rest of the tarsal plate in an attempt to enhance model performance using actual clinical metrics. Designing AI tool which function using the same logic as clinical decision making is thought to improve model explainability, which can be important for the acceptability of AI systems [38–40]. First, the image is transformed from an RGB color space [41] to an HSV color space [42]. HSV was chosen by inspection of Trachoma positive images in various color spaces, as seen in Fig 1; we selected to use the space which most accentuates the follicles to a human observer, which was primarily in the S channel of the HSV space. The follicles were further enhanced through the application the Contrast Limited Adaptive Histogram Equalization [43] algorithm to the selected HSV space as seen in Fig 2. This method was then applied to all the images and the model was retrained.

### Skilled grader overread

To mimic a conventional reading center with a tiered grading structure, all images with a predicted class of positive for TF were then graded (“overread”) by a skilled human grader different from the expert who completed the initial grading. The final grades were then compared with the consensus grade using sensitivity, specificity, and the kappa statistic. In elimination programs, a kappa of ≥ 0.7 is considered sufficient agreement to validate a field grader [44], however we note that in the seminal Global Trachoma Mapping Project (GTMP) protocol, the disease prevalence was recommended to be between 30% and 70% TF. Likewise, the most recent Tropical Data training documentation recommends a minimum prevalence of 10% for an intergrader assessment [45]. The GTMP and Tropical Data authors recognized that kappa is dependent on prevalence, such that in very low (or very high) prevalence environments the kappa threshold is harder to meet and thus the skill level of a grader or any new diagnostic tool in such an environment must be higher than what was historically considered acceptable in moderate prevalence environments [43].

### Validation and model updates

In order to externally validate our model, we utilized an independent dataset, described in [22]. We note that this dataset contains three classes: without active trachoma, TF, and trachomatous inflammation—intense (TI), the latter two of which can coexist. We utilized only the data that either did (n = 527) or did not (n = 1019) fulfil the criteria for the two signs of the WHO simplified grading system. Images that were positive for both TF and TI were included, but images that were solely positive for TI were excluded. After validation, we synthesized our data with the independent dataset and retrained the model using the network architecture and hyper-parameters from our best-performing model.

### Performance metrics

In any type of clinical classifier or binary diagnostic tool, balance must be sought between false positive and false negative results, the relative importance of which will be dictated by the specifics of the clinical scenario. Because this tool is designed for use in low TF prevalence environments, false positives have a disproportionate effect on the output of most public health interest: district level prevalence. Since the motivation of this stage is to reduce, rather than eliminate, the skilled image-evaluation burden (defined as the proportion of images requiring a skilled/human grade), if almost all normal images can be eliminated, expert graders can still be used to overread positives to improve the specificity of a positive prediction. As such, the primary metric we use to gauge model performance is recall or sensitivity, defined as the number of true positive predictions divided by the number of true positives and false negatives.

## Results

**Fig 3:**
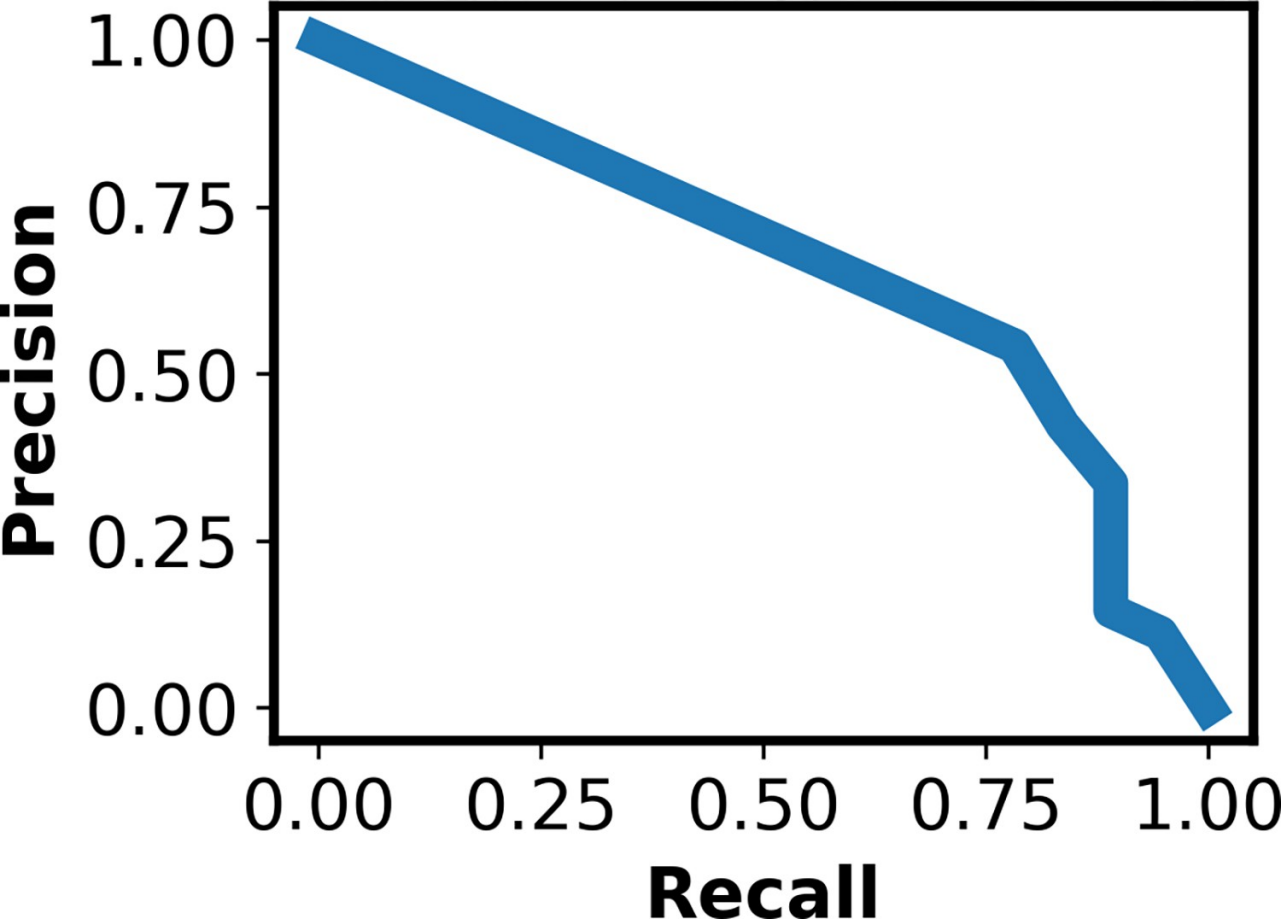
Precision-Recall Curve for Best Performing ICAPS Model: The precision-recall curve for the best performing model trained on ICAPS data with a threshold of 0.2. Precision, displayed on the y-axis, is defined as the total number of images that are correctly identified as positive for TF divided by the total number of images that are correctly identified as positive for TF plus the total number of images that are incorrectly identified as positive for TF. Recall, shown on the x-axis, is defined as the total number of images that are correctly identified as positive for TF divided by the total number of images that are correctly identified as positive for TF plus the total number of images that are incorrectly identified as negative for TF.

**Fig 4:**
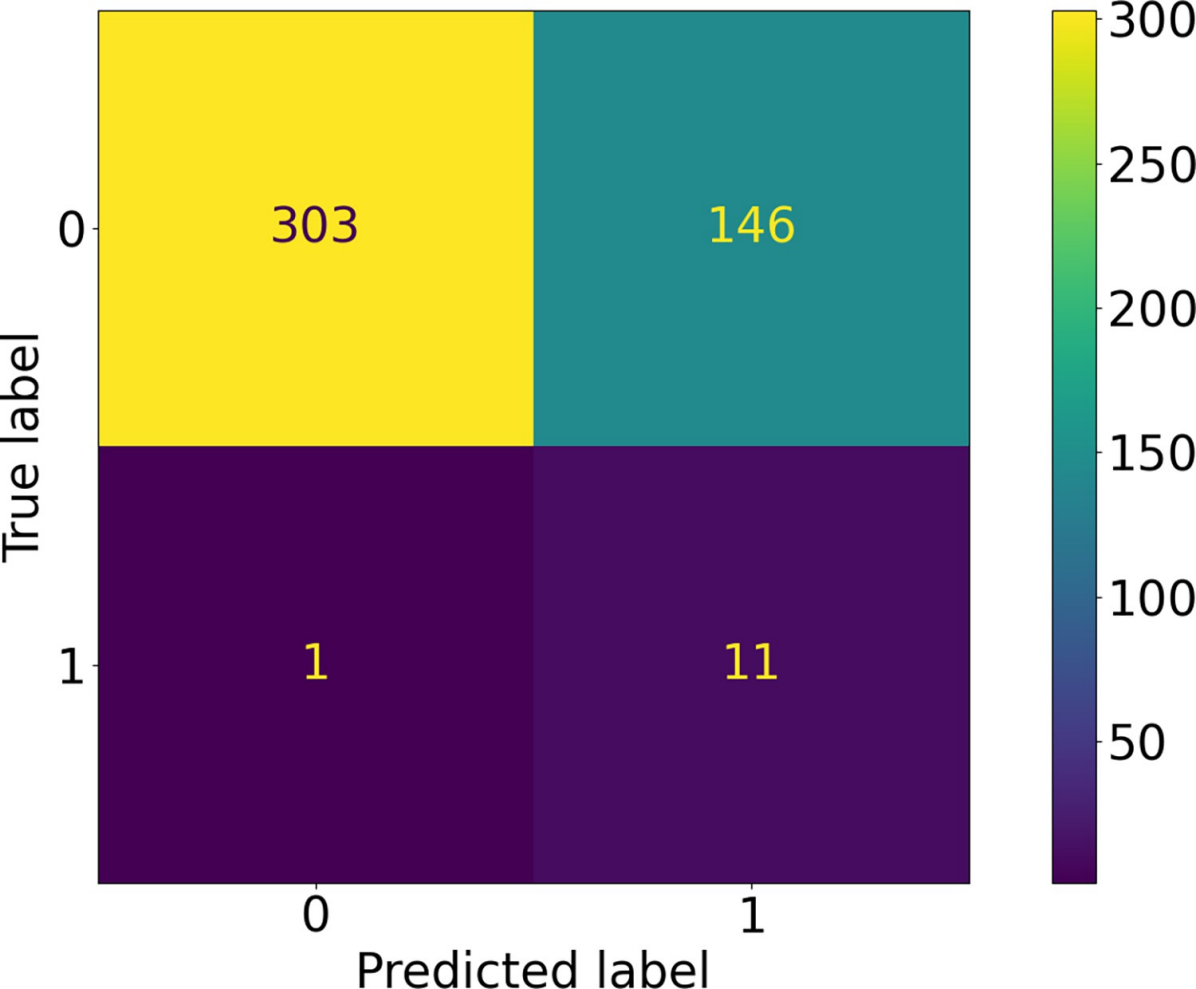
Confusion Matrix for Best Performing ICAPS Model: A comparison of predicted vs ground truth classes for the best performing model trained on ICAPS data with a threshold of 0.2. The number of images that were correctly predicted as negative for TF are displayed in the top left; images that were incorrectly classified as positive for TF are displayed in the top right; images that are incorrectly classified as negative for TF are displayed in the bottom left; images that are correctly classified as positive for TF are shown in the bottom left.

As described in Methods, we applied a number of data augmentation/oversampling techniques and transforms in various combinations on a subset of the data. In Table 1, we present the recall from 10 stochastic replicates of network training, for the top 3 performing networks; our best performing model utilized the Resnet101 architecture with the horizontal flip, perspective shift, and rotation transforms. To tune the classification threshold (i.e., the boundary between the two classes), we examined the precision-recall curve, shown in Fig 3. We set the threshold at 0.2, which gives a recall of 89%. We note that this threshold is low, which introduces the danger of false positives, but minimizes false negatives. This is illustrated in Fig 4, a confusion matrix representing the best-performing model. For this confusion matrix, class 0 represents the TF-negative class and class 1 represents the TF-positive class; the ground truth is shown on the x-axis and model predictions are shown on the y-axis. As discussed above, there were a significant number of false positives, 146.

**Table 1:** Recall values for combinations of network architecture and data augmentation techniques described in Methods.

Network Architecture/Data Augmentation	Test Recall
Resnet; flip, rotate, perspective	0.88
Resnet; flip, rotate, perspective, jitter	0.56
VGG16; flip, rotate, perspective	0.83

When the AI classifier was used as “first pass” with skilled human overread of the 157 positive images, the kappa agreement increased from 0.086 of the classifier prediction alone to 0.659. Specificity increased to 99.6% from 67.5%, though sensitivity decreased from 91.7% to 58.3%. The prevalence of TF in the test set per the ground truth grade was 2.60% (95% confidence interval (CI): 1.35%-4.50%) as compared to 1.74% (95% CI: 0.75%-3.34%) using overreads and 34.0% (95% CI: 29.7%-38.6%) with the AI classifier alone. Of note, the grading task was completed by the skilled grader in approximately 20 minutes and constituted a 66% reduction in grading burden.

**Fig 5:**
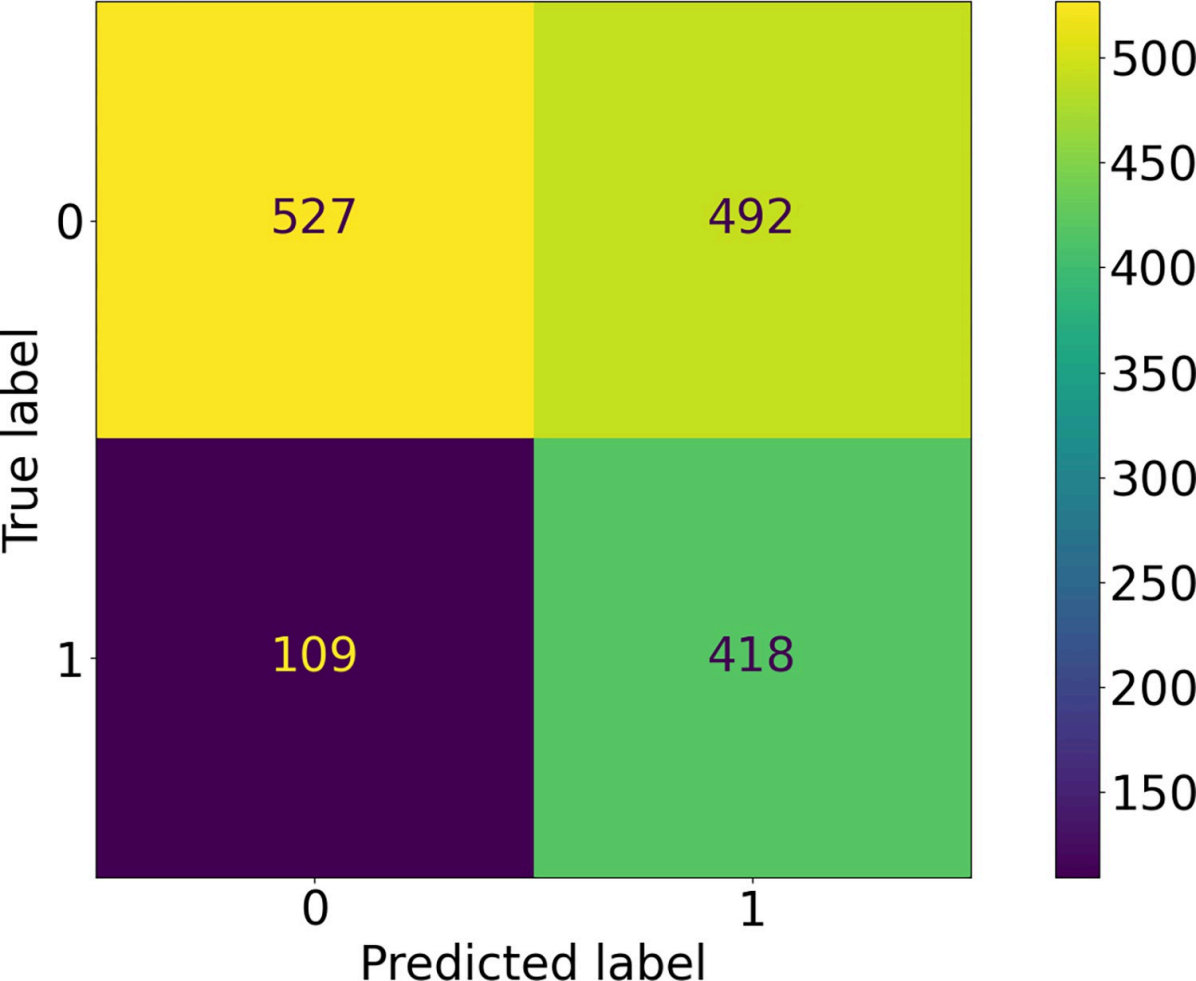
Confusion Matrix for Best Performing ICAPS Model Validated Against External Dataset. A comparison of predicted vs ground truth classes for the best performing model trained on data from ICAPS and tested with Kim et al [22] with a threshold of 0.2. The number of images that were correctly predicted as negative for TF are displayed in the top left; images that were incorrectly classified as positive for TF are displayed in the top right; images that are incorrectly classified as negative for TF are displayed in the bottom left; images that are correctly classified as positive for TF are shown in the bottom left.

**Fig 6:**
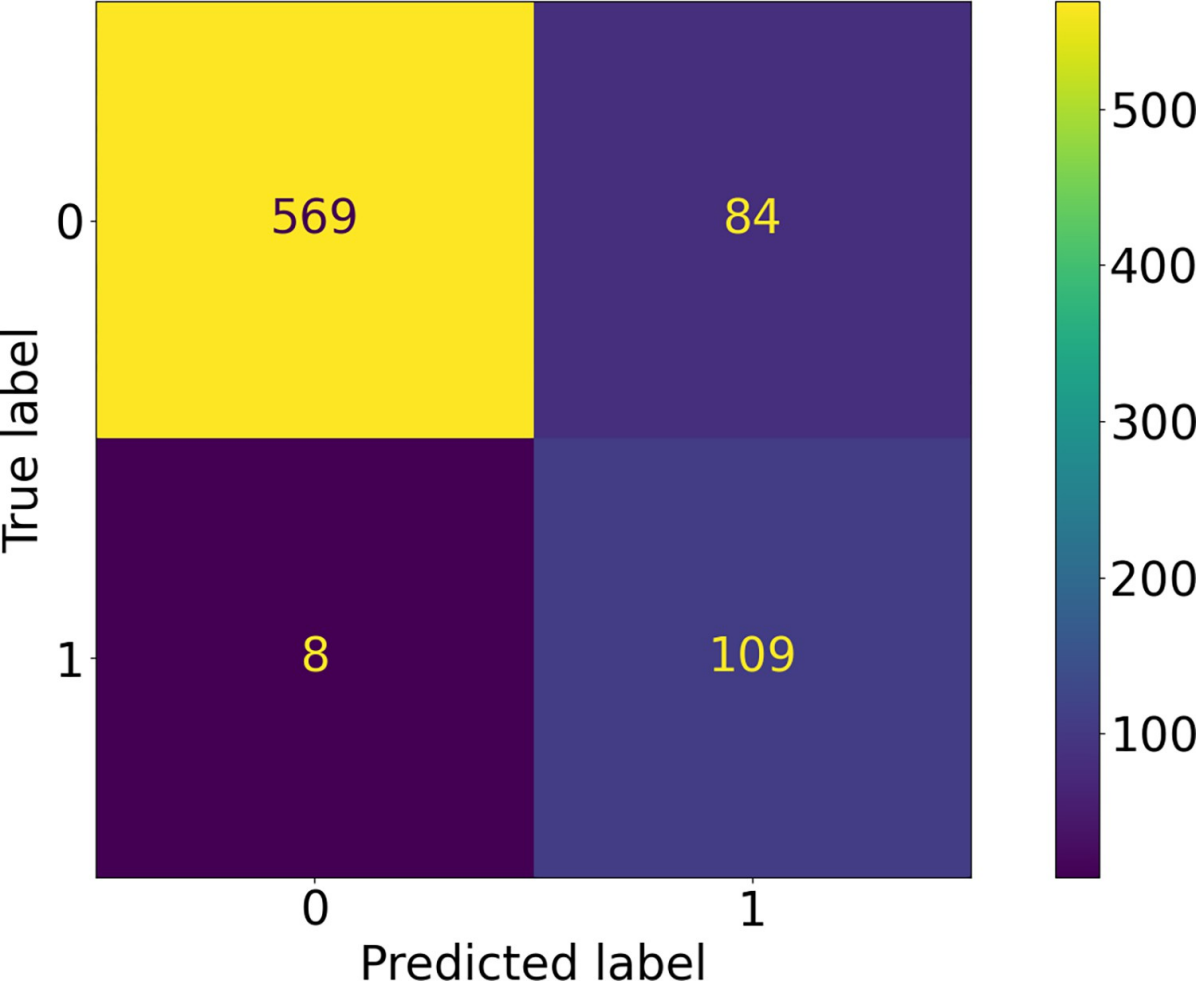
Confusion Matrix for Best Performing Combined Model. A comparison of predicted vs ground truth classes for the best performing model trained on two independent datasets with a threshold of 0.2. The number of images that were correctly predicted as negative for TF are displayed in the top left; images that were incorrectly classified as positive for TF are displayed in the top right; images that are incorrectly classified as negative for TF are displayed in the bottom left; images that are correctly classified as positive for TF are shown in the bottom left.

**Fig 7:**
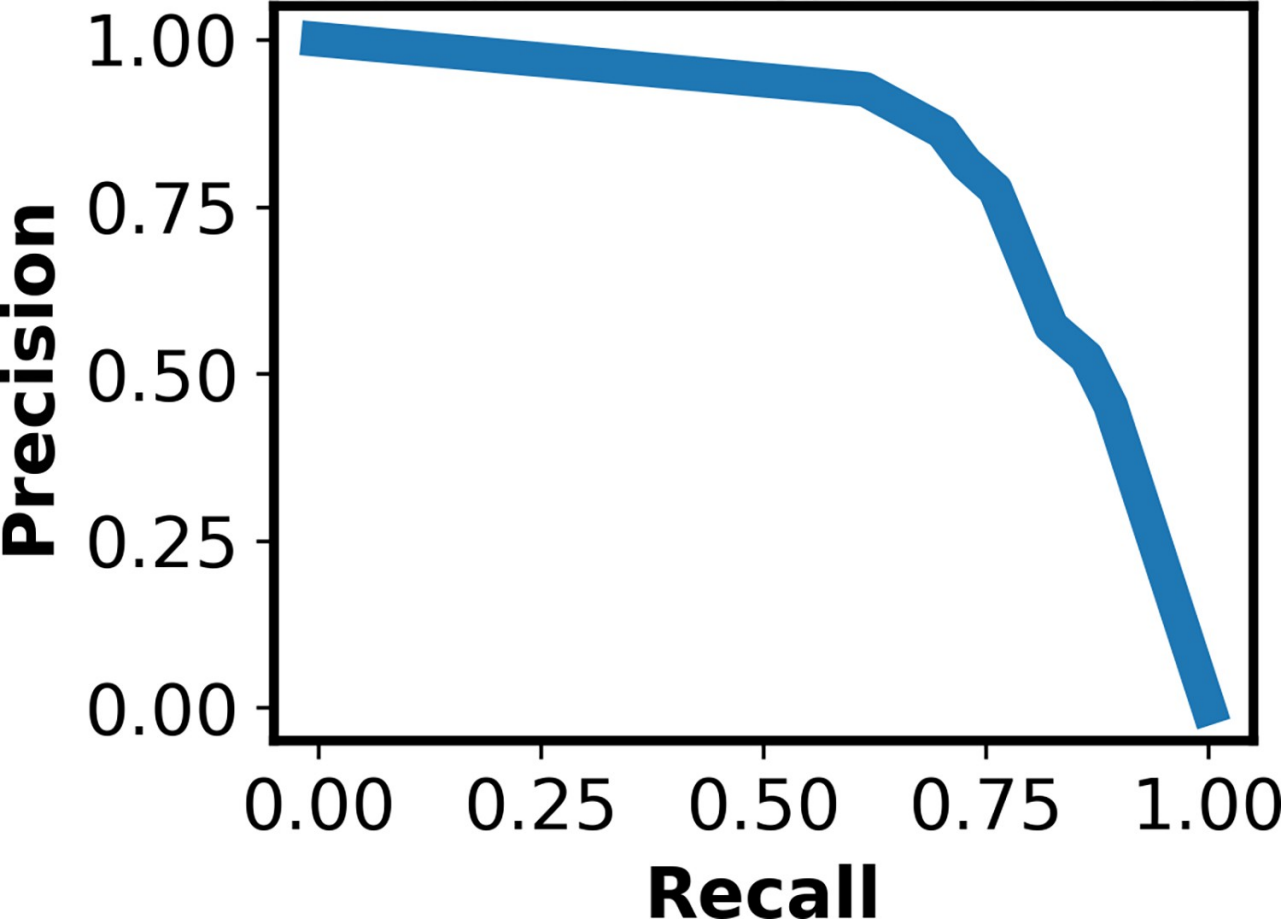
Precision-Recall Curve for Best Performing Combined Model. The precision-recall curve for the best performing model trained on two independent datasets with a threshold of 0.2. Precision, displayed on the y-axis, is defined as the total number of images that are correctly identified as positive for TF divided by the total number of images that are correctly identified as positive for TF plus the total number of images that are incorrectly identified as positive for TF. Recall, shown on the x-axis, is defined as the total number of images that are correctly identified as positive for TF divided by the total number of images that are correctly identified as positive for TF plus the total number of images that are incorrectly identified as negative for TF.

To externally validate the ICAPS-trained model, we utilized an independent dataset containing 1546 images from Ethiopia and Niger [22], illustrated in Fig 5, which generated a precision of 0.46 and a recall of 0.79. This decrease in performance is not unexpected given the paucity of TF-positive cases in our dataset. We then combined the datasets, noting that data from [22] contained images of a third class, TI, which were excluded. This resulted in a training set with 466 images that were positive for TF and 3262 images that were negative for TF, with data augmentation performed as described above; 117 images in the test set were positive for TF. This addition significantly increased the model’s performance, illustrated in Fig 6, generating a precision of 0.56 and a recall of 0.93. We show the precision-recall curve for this model in Fig 7. After skilled overread of the 193 images that were classified as positive for TF by the CNN, kappa agreement increased to 0.787 from 0.634. Specificity increased to 97.6% from 87.1% while sensitivity decreased to 78.6% from 93.1%. The prevalence of TF in the combined set per the ground truth grade was much higher than in the ICAPS data set at 15.2% (95% CI: 12.7%-17.9%) as compared to 14.0% (95% CI: 11.6%-16.7%) using overreads and 25.1% (95% CI: 22.0–28.3%) with the AI classifier alone. In this instance, the grading task was completed by the skilled grader in approximately 30 minutes and once again constituted a 75% reduction in grading burden. Detailed training/validation/test data splits are available at https://github.com/An-Cockrell/Trachoma.

Additionally, we trained the networks using the follicle enhancement technique described in methods. Notably, these models performed worse when considering the goal of minimizing false negatives, though they were more successful at reducing false positives. We compare the relative precision/recall metrics for these models in Table 2.

**Table 2:** Precision, Recall, and Kappa scores for the best-performing model architecture trained and tested on various combinations of independent datasets.

Training Data	Test Data	Precision	Recall	Kappa
ICAPS	ICAPS	0.07	0.92	0.09/0.66^+^
ICAPS	Kim et al [22]	0.46	0.79	0.26
ICAPS+Kim et al	ICAPS+Kim et al	0.56	0.93	0.63/0.79^+^
Kim et al	Kim et al	0.68	0.95	0.66
Kim et al	ICAPS	0.11	0.83	0.16
Kim et al*	Kim et al*	0.47	0.72	0.54

*denotes the dataset was modified to contain only 5% TF-positive images. ^+^ denotes that the Kappa statistic was calculated from overread images.

## Discussion

These results indicate that, given a sufficient training set, it should be possible to train an AI to detect TF with a similar accuracy to a trained expert clinician. In the current report, we relied on the AI classifier to provide a “first-pass” for a skilled grader and found an approximately 70% reduction in the grading burden with results comparable to conventional live grading, though we anticipate acceptable standalone results with future refinements as was demonstrated by retraining with an expanded external dataset. An automated tool to accurately identify TF is desirable for several reasons, including cost and efficiency of disease prevention, however, in our opinion, its greatest value would lie in the ability of the model to store knowledge/skill (the ability to diagnose TF) that is becoming increasingly hard to acquire and maintain given the decreasing prevalence of the disease. Additionally, we would like to emphasize three notable findings in Table 2: 1) the best results were achieved when using only the Kim et al. [22] dataset from Niger and Ethiopia that are characterized by a balanced number of TF-positive:TF-negative cases and high image quality with little variation in image capture technique between images; however, the high number of TF cases may not be representative of districts where TF is very low and thus, kappa may be relatively inflated. Steps must be taken to ensure systems are validated in realistic environments to mitigate spectrum bias/effect [46]; 2) models trained on a single dataset and tested on an independent dataset in general do not perform as well (see rows 2 and 5 in Table 2); 3) our combined dataset performs very well, but its performance is slightly degraded by the greater variation in image capture technique across studies.

In contrast with previous efforts to construct a Machine-Learning (ML) pipeline for the identification of TF, we did not develop a cropping procedure; however we note that there is a key qualitative difference in our images compared to theirs: the image collector in [22] wore examination gloves while our image collectors were ungloved. Upon visual inspection in alternate color space, we found the tissue underneath the thumbnail to be very similar to the epithelial tissue on the tarsal plate, which we expect would introduce difficulties into an automated cropping procedure. Despite this, we recognize the importance of maximizing the amount of useful information fed to the neural network through image standardization. As an alternative to automated cropping, we would suggest a more standardized method of image collection (e.g., distance to camera, centering of tarsal plate, etc.), and this would be easily achievable with a screen overlay on the AR-system utilized in the initial ICAPS study [47]. We note the significantly improved performance of the AI model upon training with the alternate dataset as evidence for this assertion. In addition to the use of white latex gloves to help differentiate examiner thumbnails from the subject’s tarsal plate, images utilized in [22] were typically in excellent focus and had the tarsal plate relatively centered in the image. Thus, the inclusion of this data can be considered to be predictive of what would happen if we could augment our dataset with higher-quality images. Given the wide range of features that can cause an image to be ungradable or incorrectly graded (e.g., blur, glare, presence of fingernails), we suggest that efforts be made to standardize image capture; these could include things like modifications to cell-phone camera screen, such as the inclusion of some targeting reticle/boundary in which the eyelid should be centered in the image. Future efforts to train AI classifiers to identify TF on poor quality images may be needed for final app deployment, but we believe efforts to standardize imaging should be prioritized.

We note that when transforming the images such that the clinical features used by expert graders are highlighted (the follicular enhancement transform described above), the model performance degraded. We speculate that this is due to the AI model not “learning” the clinical definition of TF, i.e., the presence of five or more follicles greater than 0.5 mm in diameter on the tarsal plate of a single eyelid. This is especially relevant in light of the recent paper from [40], describing, among other things, the dichotomous criteria ML models and physicians use to make diagnoses. While an AI model that meets or exceeds physician/expert classification performance is obviously desirable, it is more desirable to develop an AI model that meets or exceeds physician/expert classification performance while using the same methodology (identifying and counting follicles) as the expert grader, as this build confidence in the model and reduces the “black-box” sentiment of AI [38]. In future work, we will be examining pre-training methods which we believe will guide the AI model towards this goal, inspired by the work presented in [39], though without explicit image segmentation techniques.

Ultimately, we anticipate that these models (and other models of this type) will initially be used to augment, rather than replace, the capabilities of trained and/or expert image graders. While the number of false-negative diagnoses for TF was well minimized in this study, with suggestion that it will be further minimized with the addition of new data, the number of false positive diagnoses would still lead to the initiation of unnecessary pharmacologic therapy without skilled overreading. Use of these models as pre-screening tools can significantly reduce the grading burden on expert graders and allow for more rapid and wide-reaching evaluations of TF prevalence in the developing world.
